# Prevalence and predictors of persistent cognitive and psychological symptoms in non-hospitalized post-COVID-19 patients seeking care at an outpatient post-COVID-19 clinic

**DOI:** 10.3389/fpsyg.2024.1396963

**Published:** 2024-08-13

**Authors:** Gisela Claessens, Iris Gerritzen, Frits van Osch, Joop P. van den Bergh, Daan Verberne, Debbie Gach, Eric van Balen, Caroline M. van Heugten

**Affiliations:** ^1^Department of Medical Psychology, VieCuri Medical Center, Venlo, Netherlands; ^2^Department of Clinical Epidemiology, VieCuri Medical Center, Venlo, Netherlands; ^3^Department of Epidemiology, Maastricht University, Maastricht, Netherlands; ^4^Department of Internal Medicine, VieCuri Medical Center, Venlo, Netherlands; ^5^NUTRIM School of Nutrition and Translational Research in Metabolism, Maastricht University, Maastricht, Netherlands; ^6^Department of Internal Medicine, Maastricht University Medical Center +, Maastricht, Netherlands; ^7^Department of Neuropsychology and Psychopharmacology, Maastricht University, Maastricht, Netherlands; ^8^Limburg Brain Injury Center, Maastricht University, Maastricht, Netherlands

**Keywords:** COVID-19, adaptation and psychological, mental disorders, cognition, depression, anxiety, delivery of health care, outcome assessment and health care

## Abstract

**Introduction:**

There is still much uncertainty about why some people develop persistent cognitive and mental health problems after SARS-CoV-2 infection and require additional care while others do not. In this study, we investigated the cognitive and psychological outcomes of non-hospitalized post-COVID-19 patients referred to an outpatient post-COVID-19 clinic for persistent symptoms more than 3 months after infection. Additionally, we aimed to explore the influence of demographic, physical, and personal factors on these outcomes.

**Methods:**

This cross-sectional study was conducted at an outpatient post-COVID-19 clinic located at a prominent clinical teaching hospital in the Netherlands. Participants included non-hospitalized patients referred between 2020 and 2022, more than 3 months after SARS-CoV-2 infection, experiencing persistent symptoms. Main outcome measures included levels of anxiety and depression (Hospital Anxiety and Depression Scale), post-traumatic stress symptoms (PTSS) (Post-traumatic Stress Symptoms Checklist 14), and cognitive symptoms (Checklist for Cognitive and Emotional Consequences). Data analysis employed Spearman correlation and hierarchical multiple regression analyses.

**Results:**

A total of 265 patients (61% female; mean age of 51.7 ± 13.7 years) were included in the study, with an average of 7.6 ± 4.5 months following SARS-CoV-2 infection. Among them, 104 patients (40%) reported high levels of anxiety, 111 patients (43%) showed high levels depressive symptoms, and 71 patients (31%) demonstrated high levels of PTSS. Additionally, 200 patients (79%) reported experiencing more than 2 cognitive symptoms. Bivariate analyses indicated associations between psychiatric history and increased cognitive and psychological symptoms. Multivariate analyses revealed positive associations between physical symptoms and cognitive and psychological symptoms, and catastrophizing thoughts were associated with higher anxiety levels (*β* = 0.217, *p* < 0.001). Conversely, positive refocusing was associated with lower depressive symptoms (*β* = −0.325, *p* < 0.001), PTSS (*β* = −0.290, *p* < 0.001), and cognitive symptoms (*β* = −0.220, *p* < 0.001).

**Discussion:**

Among non-hospitalized COVID-19 patients seeking care for persistent symptoms, approximately one-third reported high levels of psychological symptoms, and more than three-quarter experienced cognitive symptoms. Physical symptoms, psychiatric history, and a tendency to catastrophize were identified as potential risk factors for persistent psychological and cognitive symptoms. Conversely, positive refocusing demonstrated a protective effect. These findings contribute to the understanding of long-term COVID-19 outcomes and emphasize the importance of integrating a biopsychosocial perspective into treatment approaches.

## 1 Introduction

Following a SARS-CoV-2 infection, acute symptoms may persist, leading to enduring symptoms thereby posing challenges for the (mental) healthcare system. The impact extends beyond physical symptoms such as fatigue, dyspnea, cough, and headache to encompass psychological and cognitive symptoms (Ballering et al., 2022; Surapaneni et al., 2022). Persistent psychological and cognitive symptoms include depression, anxiety, post-traumatic stress symptoms (PTSS), memory and concentration problems, slowness in thinking, and confusion (Mazza et al., 2020; Evans et al., 2021; Taquet et al., 2021; Fang et al., 2022; Houben-Wilke et al., 2022; Tabacof et al., 2022). A meta-analysis revealed that long-term neurological and neuropsychiatric symptoms were prevalent in both hospitalized and non-hospitalized patients (Premraj et al., 2022). This suggests that neither the initial severity of the SARS-CoV-2 infection nor the necessity for clinical care following infection determines the development of persistent symptoms. Since the majority of post-COVID-19 patients have not required hospitalization, with approximately 135 thousand out of 8.6 million infected individuals in the Netherlands having been admitted to hospitals between 2020 and 2022, it is evident that the non-hospitalized cohort comprises the largest patient group (Dutch National Intensive Care Evaluation, 2023; World Health Organization, 2023).

Identified risk factors for persistent psychological and cognitive symptoms include increasing age, female sex, persistent physical symptoms, medical comorbidities, low income, minority race/ethnicity, and psychiatric history (Michelen et al., 2021; Taquet et al., 2021; Righi et al., 2022; Abramoff et al., 2023; Zakia et al., 2023). Similar patterns are observed in other infectious diseases such as Q fever and Lyme disease, where a subset of individuals experiences persistent symptoms (Reukers et al., 2020; Hündersen et al., 2021). Personal factors, such as pre-existing emotional problems or psychiatric history, along with specific coping styles, predict long-term psychological functioning in these patients (Hill and Frost, 2022; Huiberts et al., 2022). To our knowledge, no studies have explored the combined influence of personal and physical factors on cognitive and psychological functioning among non-hospitalized post-COVID-19 patients. Additionally, few studies have addressed psychological and cognitive functioning of non-hospitalized post-COVID-19 patients necessitating further care for persistent symptoms (Abramoff et al., 2023). Identifying risk factors associated with cognitive and psychological functioning is crucial for understanding the etiology of persistent symptoms, facilitating the prediction of recovery processes, and the formulation of new treatment strategies for managing post-COVID-19 healthcare. Noteworthy for clinicians is the identification of modifiable personal factors, such as coping, which could be targeted in treatment to reduce symptoms and enhance patient resilience (Lemogne et al., 2023).

This study aimed (1) to examine psychological and cognitive functioning of non-hospitalized patients seeking care for long-term symptoms after SARS-CoV-2 infection; and (2) to investigate the independent contribution of demographic, physical, and personal factors on cognitive and psychological functioning post-COVID-19.

## 2 Materials and methods

### 2.1 Design and participants

This study employed a single-center, cross-sectional design. The study focused on patients who had experienced a following SARS-CoV-2 infection and sought care at an outpatient post-COVID-19 clinic after being referred by a general practitioner or medical specialist. Initially, referral criteria included patients with persistent fatigue, diminished fitness, and pulmonary symptoms such as exertional dyspnea, thoracic symptoms, or other symptoms, suspected of a previous SARS-CoV-2 infection. Subsequently, this was revised to include only those with a confirmed SARS-CoV-2 infection more than 3 months ago. Inclusion criteria for the study involved having a proficient command of the Dutch language to complete questionnaires and a willingness to complete the questionnaires. Exclusion criteria were age under 18, hospital admission following SARS-CoV-2 infection, and time since SARS-CoV-2 infection less than 3 months.

### 2.2 Procedure

Data were collected during visits to the outpatient post-COVID-19 clinic at a prominent clinical teaching hospital in the Netherlands, serving approximately 280,000 inhabitants. After referral by the general practitioner or medical specialist, patients concurrently visited the pulmonologist and internist. Standardized cognitive and psychological questionnaires [Hospital Anxiety and Depression Scale (HADS), Post-traumatic Stress Symptoms Checklist 14 (PTSS-14), and Checklist for Cognitive and Emotional Consequences (CLCE-24)] were provided during or after consultation (Zigmond and Snaith, 1983; van Heugten et al., 2007; Twigg et al., 2008). Patients were requested to return completed questionnaires using an enclosed envelope to the Medical Psychology department. Patients not proficient in Dutch or unwilling to complete the questionnaires did not receive them. Following questionnaire return, a psychologist contacted patients, conveyed the results, and assessed the need for additional psychological treatment for coping with cognitive and/or emotional symptoms. Treatment was recommended for patients experiencing difficulties dealing with such symptoms, initiated or exacerbated after SARS-CoV-2 infection, impacting daily life functioning. Over time, clinical practice prompted the inclusion of the Four-Dimensional Symptom Questionnaire (4DSQ) and Cognitive Emotion Regulation Questionnaire (CERQ) to gain additional insight into physical symptoms and coping styles (Terluin, 1996; Garnefski et al., 2001).

Study data were retrieved from medical records and stored in a COVID-specific hospital database in Castor EDC (Castor, 2019). The study was conducted in accordance with the Medical Ethical Committee of Maastricht University Medical Center+, falling beyond the scope of the Medical Research Involving Human Subjects Act (WMO) (2021–3,059). The study was approved by the local institutional review board. Patients were informed about potential research use of their clinical data and had the option to opt out. Data collection occurred between July 1st 2020, and January 1st, 2023.

### 2.3 Measures

Demographic characteristics included age, sex, and time between SARS-CoV-2 infection and the post-COVID-19 clinic visit.

The HADS is a self-assessment tool assessing depression and anxiety symptoms (Zigmond and Snaith, 1983). It comprises anxiety (HADS-anxiety) and depression (HADS-depression) subscales, each with seven items. Responses, on a four-point Likert scale (0–3), yield subscale scores ranging from 0 to 21; higher scores indicate more symptoms of anxiety or depression. A cut-off score of ≥8 per subscale indicated clinically relevant symptoms of anxiety or depression (Herrmann, 1997).

The PTSS-14 is a screening questionnaire identifying patients at risk of post-traumatic stress disorder (PTSD) (Twigg et al., 2008). It features 14 items on a seven-point Likert scale (1–7), with scores ranging from 14 to 98; higher scores indicate more PTSS. Possible traumatic events were assessed by evaluating nightmares, anxiety and panic attacks, severe pain, and breathing difficulties and feelings of choking during the period of SARS-CoV-2 infection. The questions were slightly modified to specify the illness period of the SARS-CoV-2 infection. A cut-off score of ≥45 indicated high levels of PTSS (Twigg et al., 2008).

The CLCE-24 comprises 24 questions screening for cognitive and emotional symptoms, with 13 questions specifically assessing the absence or presence of cognitive symptoms (CLCE-cognition) and 1 item assessing the presence or absence of fatigue (CLCE-fatigue). Higher scores indicate more cognitive problems experienced in daily life (range 0–13) (van Heugten et al., 2007). We used a cutoff >2 based on mean of 1.9 (standard deviation = 1.9) in healthy controls (van Rijsbergen et al., 2015).

The 4DSQ measures the tendency to experience distress, somatization, depression, and anxiety (Terluin, 1996). For this study, only data from the somatization subscale, assessing physical symptoms over the past weeks, were used. The somatization subscale comprises 16 items (scores range 0–32), with scores between 11 and 20 indicating moderate physical symptoms and scores >20, used as cut-off in this study, indicating severe physical symptoms (Terluin et al., 2008).

The CERQ measures patients’ generally used cognitive coping strategies (Garnefski et al., 2002). It is a 36-item questionnaire featuring nine conceptually distinct subscales (Garnefski et al., 2001). Items are scored on a five-point Likert scale (1–5), with subscale scores ranging from 4 to 20. Higher scores indicated more frequent use of the coping strategy. Normative data was obtained from the CERQ manual (Garnefski et al., 2002).

### 2.4 Statistical analysis

The study population was described using means (SD) and medians (IQR). Numbers and percentages were reported for binary variables and variables with cut-off values. We presented both raw scores and *z*-scores of the CERQ.

Bivariate analyses, using Spearman correlation coefficients with Bonferroni correction for multiple comparisons (0.05/10 personal factors), were used to calculate associations between personal factors (i.e., psychiatric history and CERQ subscales) and cognitive and psychological outcomes. Four hierarchical multiple regression analyses were conducted to assess associations between demographic, physical, and personal factors and psychological and cognitive outcomes. Dependent variables were scores on HADS-anxiety, HADS-depression, PTSS-14, and CLCE-cognition. Age, sex, and time since SARS-CoV-2 infection were added as independent variables to the first block, and physical factors (4DSQ score) to the second block. Personal factors (CERQ subscales) found significant in bivariate analyses were added to the third block. Psychiatric history was omitted in the multivariate analyses due to missing data and lack of power. After applying the Bonferroni correction (0.05/4), the alpha level was set at 0.01 for multivariate analyses. Regression analysis assumptions were met for each model. IBM SPSS Statistics, version 26, was used for data analysis.

## 3 Results

### 3.1 Participants

**Figure 1:**
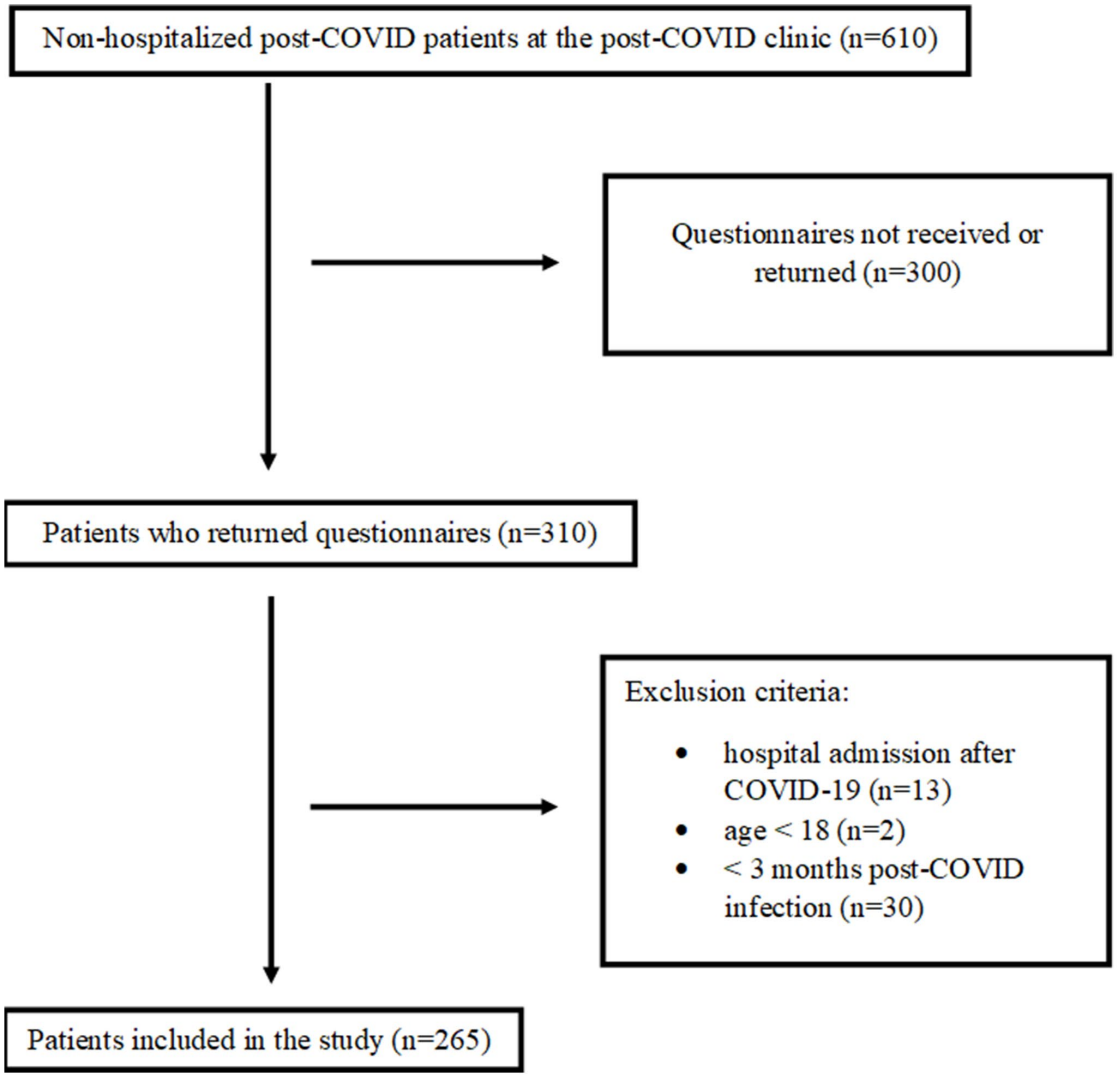
Flowchart for patient inclusion.

Figure 1 indicates that 610 patients were referred to the post-COVID-19 clinic during the study period. Of these patients, 310 (51%) returned the questionnaires, and 45 patients were excluded. In total, data from 265 patients were used in this study.

Table 1 presents the characteristics of the patients included in this study. Approximately 1 in 5 patients had a known psychiatric history (22%). One third of the patients (36%) were indicated for psychological treatment for their symptoms and visited the Medical Psychology department.

**Table 1:** Patient characteristics (*n* = 265).

Variable	Mean (SD)/Median (IQR)/*n* (%)
Sex, male	103 (39)
Age in years	
Mean (SD)	51.7 (13.7)
Median (IQR)	53.0 (17.0)
Time since infection in months	
Mean (SD)	7.6 (4.5)
Median (IQR)	6.1 (4.9)
3–6 months	122 (46)
>6 months	143 (54)
Psychological history (yes)	58 (22)
Missing	36 (14)
Indication for psychological treatment (yes)	96 (36)

Table 2 outlines the scores on the physical and personal variables and outcome measures. Of the patients, 95% reported the presence of fatigue symptoms on the CLCE-fatigue. More than half of the patients (53%) who completed the 4DSQ reported scores above the cut-off. Approximately, between 30 and 40% scored above cut-off values on the HADS-anxiety, HADS-depression, and PTSS-14. Over three quarters of patients (79%) reported more than two cognitive symptoms on the CLCE-cognition. The most common cognitive symptoms were mental slowness (72%), difficulties in paying attention (74%), and remembering new information (75%).

**Table 2:** Descriptive of physical and personal variables and outcome measures (*n* = 265).

Measures and domains	Range in data	*n*	Mean (SD)	Median (IQR)	*n* (%) above/below cut-off
*Physical and personal variables*					
4DSQ	1–32	151	13.7 (7.0)	13.0 (10.0)	140 (53)
CLCE-fatigue		262			248 (95)
CERQ					
Self-blame	0–19	149	6.4 (3.3)	5.0 (4.0)	2 (1) * 5 (3) †
Acceptance	0–20	149	11.3 (3.7)	11.0 (5.0)	7 (5) * 5 (3) †
Rumination	0–19	149	9.4 (4.0)	9.0 (6.0)	2 (1) * 5 (3) †
Positive refocusing	0–20	149	11.2 (4.2)	11.0 (6.0)	2 (1) * 15 (10) †
Planning	0–20	149	12.7 (4.2)	13.0 (6.0)	7 (5) * 0 †
Positive reappraisal	0–20	149	10.8 (4.5)	10.0 (7.0)	11 (7) * 0 †
Putting thing in perspective	0–20	149	12.5 (4.6)	13.0 (7.0)	2 (1) * 7 (5) †
Catastrophizing	0–18	149	6.0 (2.9)	5.0 (3.0)	2 (1) * 13 (9) †
Other blame	0–18	149	5.4 (2.6)	4.0 (2.0)	2 (1) * 6 (4) †
*Outcome measures*					
HADS-anxiety	0–18	259	6.7 (4.3)	6.0 (7.0)	104 (40)
HADS-depression	0–20	259	7.0 (4.6)	7.0 (7.0)	111 (43)
PTSS-14	14–74	233	35.8 (14.5)	34.0 (22.0)	71 (31)
CLCE-cognition	0–13	254	5.8 (3.5)	6.0 (6.0)	200 (79) ‡

* <2 SD as compared to normative data. † > 2 SD as compared to normative data. ‡ > 2 cognitive symptoms.

### 3.2 Associations and predictors of anxiety

Bivariate analyses revealed that six coping subscales were significantly associated with higher anxiety scores: a higher tendency toward self-blame (*r* = 0.320, *p* < 0.001), rumination (*r* = 0.484, *p* < 0.001), catastrophizing (*r* = 0.411, *p* < 0.001), and other-blame (*r* = 0.298, *p* < 0.001), and a lower tendency toward positive refocusing (*r* = −0.315, *p* < 0.001) and positive reappraisal (*r* = −0.228, *p* < 0.001). Multivariate analyses showed that demographic factors explained 5.2% of the variance in anxiety (step 1). Physical symptoms explained an additional 35.8% of the variance in anxiety (*F* change = 86.222, *p* < 0.001) (step 2). Personal factors (coping) explained an additional 15.2% of the variance in anxiety (*F* change = 7.862, *p* < 0.001). In the final model, more physical symptoms (*β* = 0.482, *p* < 0.001) and a higher tendency toward catastrophizing (*β* = 0.217, *p* < 0.01) were significantly associated with higher anxiety scores (*R*^2^ = 0.562) (Table 3).

**Table 3:** Associations and predictors of anxiety and depression (*n* = 147).

Outcome	HADS-anxiety				HADS-depression			
	Bivariate (rs)	Multivariate (β)			Bivariate (rs)	Multivariate (β)		
Predictor		Step 1	Step 2	Step 3		Step 1	Step 2	Step 3
Age		−0.168	−0.031	−0.033		−0.127	−0.019	−0.013
Sex		−0.038	−0.046	−0.015		−0.081	−0.088	−0.062
Time since infection		0.156	0.127	0.053		0.223†	0.199†	0.167
Physical symptoms		NE	0.615†	0.482†		NE	0.479†	0.367†
Psychiatric history	0.142				0.216*			
Self-blame	0.320*	NE	NE	0.059	0.246*	NE	NE	0.059
Acceptance	0.076	NE	NE	NE	0.089	NE	NE	NE
Rumination	0.484*	NE	NE	0.093	0.441*	NE	NE	0.117
Positive Refocusing	−0.315*	NE	NE	−0.118	−0.406*	NE	NE	−0.325†
Planning	−0.002	NE	NE	NE	0.009	NE	NE	NE
Positive Reappraisal	−0.228*	NE	NE	−0.178	−0.208	NE	NE	NE
Putting into Perspective	−0.091	NE	NE	NE	−0.156	NE	NE	NE
Catastrophizing	0.411*	NE	NE	0.217†	0.406*	NE	NE	0.137
Other-blame	0.298*	NE	NE	0.005	0.232*	NE	NE	−0.081
*R* ^2^		0.052	0.410	0.562		0.068	0.285	0.454
Adjusted *R*^2^		0.032	0.393	0.530		0.048	0.265	0.418

NE, not entered. *Bivariate analyses: *p* ≤ 0.005. †Multivariate analyses: *p* ≤ 0.01.

### 3.3 Associations and predictors of depression

Bivariate analyses showed that psychiatric history and five coping subscales were significantly associated with higher depression scores: psychiatric history (*r* = 0.216, *p* < 0.001), a higher tendency toward self-blame (*r* = 0.246, *p* < 0.001), rumination (*r* = 0.441, *p* < 0.001), catastrophizing (*r* = 0.406, *p* < 0.001), and other-blame (*r* = 0.232, *p* < 0.001), and a lower tendency toward positive refocusing (*r* = −0.406, *p* < 0.001). Multivariate analyses demonstrated that demographic factors explained 6.8% of the variance in depression (step 1). Physical factors explained an additional 21.7% of the variance in depression (*F* change = 43.128, *p* < 0.001) (step 2). Personal factors (coping) explained an additional 16.9% of the variance in depression (*F* change = 8.477, *p* < 0.001) (step 3). In the final model, more physical symptoms (*β* = 0.367, *p* < 0.001) and a lower tendency toward positive refocusing (*β* = −0.325, *p* < 0.001) were the only independent factors significantly associated with higher depression scores (*R*^2^ = 0.454) (Table 3).

### 3.4 Associations and predictors of PTSS

Bivariate analyses showed that psychiatric history and four coping subscales were significantly associated with higher PTSS scores: psychiatric history (*r* = 0.211, *p* < 0.001), a higher tendency toward rumination (*r* = 0.481, *p* < 0.001), catastrophizing (*r* = 0.376, *p* < 0.001), and other-blame (*r* = 0.266, *p* < 0.01), and a lower tendency toward positive refocusing (*r* = −0.354, *p* < 0.001). Multivariate analyses demonstrated that demographic factors explained 6.4% of the variance in PTSS (step 1). Physical factors explained an additional 30.7% of the variance in PTSS (*F* change = 61.539, *p* < 0.001) (step 2). Personal factors (coping) explained an additional 12.8% of the variance in PTSS (*F* change = 7.779, *p* < 0.001) (step 3). In the final model, more physical symptoms (*β* = 0.428, *p* < 0.001) and a lower tendency toward positive refocusing (*β* = −0.290, *p* < 0.001) were the only independent variables significantly associated with higher PTSS scores (*R*^2^ = 0.499) (Table 4).

**Table 4:** Associations and predictors of PTSS (*n* = 131) and cognitive symptoms (*n* = 144).

	PTSS-14				CLCE-cognition			
	Bivariate (rs)	Multivariate (β)			Bivariate (rs)	Multivariate (β)		
		Step 1	Step 2	Step 3		Step 1	Step 2	Step 3
Age		−0.097	−0.018	−0.002		−0.105	−0.008	0.004
Sex		−0.011	−0.063	−0.033		0.032	0.022	0.048
Time since infection		0.234†	0.188†	0.142		0.149	0.126	0.110
Physical symptoms		NE	0.564*	0.428†		NE	0.477†	0.444†
Psychiatric history	0.211*				0.210*			
Self-blame	0.176	NE	NE	NE	0.009	NE	NE	NE
Acceptance	0.162	NE	NE	NE	0.049	NE	NE	NE
Rumination	0.481*	NE	NE	0.215	0.230*	NE	NE	0.024
Positive Refocusing	−0.354*	NE	NE	−0.290†	−0.267*	NE	NE	−0.220†
Planning	0.019	NE	NE	NE	0.087	NE	NE	NE
Positive Reappraisal	−0.193	NE	NE	NE	−0.091	NE	NE	NE
Putting into Perspective	−0.115	NE	NE	NE	−0.090	NE	NE	NE
Catastrophizing	0.376*	NE	NE	0.004	0.179	NE	NE	NE
Other-blame	0.266*	NE	NE	0.018	0.059	NE	NE	NE
*R* ^2^		0.064	0.371	0.499		0.034	0.252	0.300
Adjusted *R*^2^		0.042	0.351	0.466		0.014	0.231	0.269

NE, not entered. *Bivariate analyses: *p* ≤ 0.005. †Multivariate analyses: *p* ≤ 0.01.

### 3.5 Associations and predictors of cognitive symptoms

According to bivariate analyses, psychiatric history (*r* = 0.210, *p* < 0.001), a higher tendency toward rumination (*r* = 0.230 *p* = 0.006), and a lower tendency toward positive refocusing (*r* = −0.267, *p* = 0.001) were significantly associated with more cognitive symptoms. Multivariate analyses showed that demographic factors explained 3.4% of the variance in cognitive symptoms (step 1). Physical factors explained an additional 21.8% of the variance in cognitive symptoms (*F* change = 40.463, *p* < 0.001) (step 2). Personal factors (coping) explained an additional 4.8% of the variance in cognitive symptoms (*F* change = 4.675, *p* = 0.011) (step 3). In the final model, more physical symptoms (*β* = 0.444, *p* < 0.001) and a lower tendency toward positive refocusing (*β* = −0.220, *p* = 0.003) were significantly associated with more cognitive symptoms (*R*^2^ = 0.330) (Table 4).

## 4 Discussion

The findings of our study revealed prevalence rates of 40% for anxiety, 42% for depression, 31% for PTSS, and 79% for more than two cognitive symptoms in non-hospitalized patients more than 3 months following SARS-CoV-2 infection. These patients sought care at an outpatient post-COVID-19 clinic. Patients with a psychiatric history reported more depressive symptoms, PTSS, and cognitive symptoms. After accounting for demographic factors, both physical and personal factors explained additional variance in outcomes. More physical symptoms were associated with increased cognitive and psychological symptoms. A greater tendency toward catastrophizing correlated significantly with higher levels of anxiety, while a higher tendency toward positive refocusing was associated with lower levels of depressive symptoms, PTSS, and cognitive symptoms.

The prevalence rates for psychological symptoms observed in this study exceeded those in the general population in the Netherlands pre-COVID, with reported prevalence rates of 9, 15, and 4% for anxiety, depression, and PTSS, respectively (Bronner et al., 2009; ten Have et al., 2023). Furthermore, around one-third of the patients were clinically indicated for psychological treatment. It should be noted that patients in the present study actively sought help in a specialized post-COVID-19 outpatient clinic for persistent physical symptoms following SARS-CoV-2 infection and actively answered questionnaires after the visit, leading to a selected study population. Nevertheless, a growing body of research highlights the increased prevalence of anxiety, depression, and PTSS in the post-acute and chronic phases following SARS-CoV-2 infection, even among non-hospitalized patients (Houben-Wilke et al., 2022; Righi et al., 2022; Zakia et al., 2023). In addition to persistent psychological symptoms, the study showed that almost 80% of patients reported persistent cognitive symptoms following SARS-CoV-2 infection, in line with previous research (Surapaneni et al., 2022). A meta-analysis highlighted fatigue, cognitive symptoms (brain fog, memory and attention problems), and sleep disturbances as prevalent problems 3 months post-COVID-19 infection, affecting almost one-third of the patients (Premraj et al., 2022). However, subjective cognitive symptoms do not necessarily indicate the presence of cognitive impairments. Among formerly hospitalized COVID-19 patients, Klinkhammer et al. (2023) found that 8–10 months post-discharge, 62% of ICU and general ward survivors reported three or more cognitive complaints, whereas standard neuropsychological testing revealed cognitive dysfunction in only 12% of patients. Discrepancies between subjective cognitive symptoms and cognitive impairment have been demonstrated across various patient populations, including stroke (van Rijsbergen et al., 2014), Lyme disease (Berende et al., 2019), and psychiatry (Groenman et al., 2022). Nevertheless, it should be noted that a lack of cognitive impairment in formal neuropsychological testing does not exclude the possibility of increased cognitive difficulties in daily life.

As anticipated, we found a significant association between physical symptoms and increased cognitive and psychological symptoms post-COVID, which is in line with Righi et al. (2022), showing a positive association between persistence of self-reported physical symptoms 9 months following SARS-CoV-2 infection and psychological distress. While the direction of influence remains unclear, it is plausible that physical symptoms contribute to cognitive and psychological symptoms, or vice versa. Symptoms of anxiety, depression, and PTSS often include physical symptoms, and patients with cognitive symptoms may experience cognitive overload, triggering a stress response with physical symptoms (American Psychiatric Association, 2013; Chu et al., 2023). Additionally, while studies have found varying associations between cognitive testing and depressive symptoms following SARS-CoV-2 infection (Kupferschmitt et al., 2023; Morawa et al., 2023), there is potential overlap between subjective perceptions of cognitive symptoms and depression, which could introduce bias due to concurrent depression. Similar associations have been observed in other patient populations, such as stroke (Nijsse et al., 2017), and among COVID-19 ICU survivors (Fjone et al., 2024). According to the DSM-V, decreased concentration is considered one of the symptoms of depression, alongside depressed mood and/or loss of interest or pleasure. Cognitive symptoms have been linked to increased depressive symptoms, greater reported functional impairment, and a decreased likelihood of returning to full-time employment post-COVID-19 (Jaywant et al., 2024). Furthermore, fatigue is frequently reported as a persistent symptom following SARS-CoV-2 infection, impacting both mood and cognitive functioning (Klinkhammer et al., 2024).

The present study underlines the importance of personal factors, including psychiatric history and coping, on psychological and cognitive outcomes. The association between psychiatric history and psychopathology has been shown previously (De Lorenzo et al., 2020; Mazza et al., 2020; Özdin and Bayrak Özdin, 2020; Huarcaya-Victoria et al., 2023), suggesting that individuals with past psychiatric illnesses may be more vulnerable to developing persistent symptoms. Coping strategies were associated with cognitive and psychological symptoms in the expected direction (Garnefski et al., 2001). Theoretically maladaptive strategies (rumination, catastrophizing, and blaming self and others) were linked to more symptoms, while theoretically adaptive strategies (positive reappraisal and positive refocusing) were associated with fewer symptoms. Among these coping strategies, only positive refocusing and catastrophizing were independently associated with outcomes, even after considering demographic and physical factors. While no studies have specifically explored the impact of coping on psychological and cognitive symptoms in post-COVID-19 patients, coping, along with other personal factors such as personality characteristics and resilience, has been linked to well-being and distress during the COVID pandemic in the general population (Zager Kocjan et al., 2021; Starcevic and Janca, 2022). Moreover, studies have consistently shown the influence of coping strategies on well-being in infectious diseases like Lyme disease and chronic illnesses such as multiple chronic conditions (Cheng et al., 2020; Huiberts et al., 2022). This underscores the importance of incorporating personal factors as risk factors when predicting outcomes.

### 4.1 Study limitations and strengths

One limitation is the use of regular clinical data, resulting in some missing variables. Missing data also occurred because not all variables were collected throughout the complete study period. No drop-out analysis was conducted because, in accordance with Dutch medical ethics regulations, if participants have not actively consented to the use of their data for research purposes—such as by not returning the questionnaires—this data may not be used or reported. Despite this limitation, the study included a large number of patients, ensuring sufficient power. Additionally, the absence of a specific questionnaire to assess COVID-19 symptomatology is acknowledged. However, many symptoms captured by the 4DSQ, including shortness of breath and muscle aches, overlap with physical symptoms post-COVID-19 (World Health Organization, 2022). Finally, we assessed cognitive symptoms using a questionnaire rather than cognitive testing. The data were collected as part of standard care. Cognitive testing has not been part of standard care for patients with post-COVID-19 syndrome.

A notable strength of this study lies in the inclusion of non-hospitalized patients facing challenges in their daily lives due to persistent post-COVID-19 symptoms, actively seeking additional care, thereby representing a substantial patient population. Within this cohort, we explored both physical and psychosocial risk factors—a distinctive approach that opens important avenues for future research and holds implications for clinical practice.

### 4.2 Future research and recommendations for clinical practice

Future research on persistent cognitive and psychological symptoms post-COVID-19 should include psychosocial factors, such as coping in addition to biomedical factors. From a cognitive-behavioral perspective it can be hypothesized that both cognitive coping (thoughts and beliefs) and behavioral coping influence outcomes. Exploring whether adding behavioral coping enhances prediction models of persistent symptoms is interesting, as cognitive coping may precede behavioral coping, with catastrophizing potentially leading to avoidance behavior, decreased activity levels, and lower quality of life (Vlaeyen and Linton, 2000). Additionally, it would be interesting to assess not only symptoms as outcomes but also levels of participation. More specifically, inability to work is common in patients admitted to a rehabilitation clinic, with almost 50% of patients reporting at least 100 days of sick leave in the past year (Kupferschmitt et al., 2023). Inability to work can have a multifaceted impact on individuals, affecting their financial, physical, emotional, and social well-being. Identifying which symptoms, including cognitive, psychological, and physical symptoms, as well as personal and social factors, are associated with participation levels can provide important insights for treatment (World Health Organization, 2001).

The study emphasizes the need to shift from a purely biomedical to a more comprehensive biopsychosocial approach in clinical practice, focusing on alternative risk factors. This approach allows for more precise referrals to psychological treatment. While it is not possible or desirable for everyone to receive treatment, it is crucial that individuals experiencing symptoms disrupting daily life do receive treatment. At present, there is no evidence-based treatment for persistent symptoms post-COVID-19. Tailoring interventions to address individual needs and incorporating modifiable factors such as coping strategies, could be beneficial. Cognitive-behavioral therapies, including second or third-wave approaches such as cognitive-behavioral therapy and acceptance and commitment therapy, have proven effective in enhancing adaptive thinking patterns and behaviors. These interventions contribute to improving psychological flexibility and enhancing the quality of life in patients with chronic illnesses (Gregg et al., 2007; Hind et al., 2014; Feldmann et al., 2021). Such interventions merit consideration for addressing post-COVID-19 symptoms. Notably, for post-COVID-19 fatigue, cognitive-behavioral therapy has shown effectiveness compared to standard care, offering a potential intervention avenue (Kuut et al., 2023).

## 5 Conclusion

A third of non-hospitalized COVID-19 patients seeking outpatient post-COVID-19 care experience persistent psychological symptoms, while three-quarter deal with cognitive symptoms. Physical symptoms, psychiatric history, high catastrophizing thoughts, and low positive refocusing are associated with long-term symptoms. The study sheds light on the mental health status of non-hospitalized patients with prolonged symptoms after COVID-19, emphasizing the potential profound impact on cognitive and psychological functioning. Recognizing at-risk patients with persistent symptoms can lead to better-tailored referrals and treatment, improving cognitive and psychological well-being, and reducing healthcare costs.

## Data availability statement

The raw data supporting the conclusions of this article will be made available by the authors, without undue reservation.

## Ethics statement

The studies involving humans were approved by Medical Ethical Committee of Maastricht University Medical Center+. The studies were conducted in accordance with the local legislation and institutional requirements. The ethics committee/institutional review board waived the requirement of written informed consent for participation from the participants or the participants’ legal guardians/next of kin. This study is part of a larger study in which also hospitalized patients were included at hospital admission and follow up. Given the observational nature of the study, coupled with the urgency of collecting baseline data in the acute setting, the Medical Ethical Committee of Maastricht University Medical Centre granted a waiver of informed consent. Subsequently, a waiver for medical ethical review was obtained for follow-up data collection. Patients attending the outpatient clinic were informed about the use of their regularly collected clinical data for research and given the option to opt out (approval number: METC 2021-3059).

## Author contributions

GC: Writing – review & editing, Writing – original draft, Methodology, Formal analysis, Conceptualization. IG: Writing – review & editing, Data curation. FO: Formal analysis, Data curation, Writing – review & editing, Methodology, Funding acquisition. JB: Writing – review & editing. DV: Writing – review & editing, Data curation. DG: Writing – review & editing. EB: Writing – review & editing, Conceptualization. CH: Writing – review & editing, Supervision, Methodology, Funding acquisition, Conceptualization.
